# Production of Recombinant Horseradish Peroxidase in an Engineered Cell-free Protein Synthesis System

**DOI:** 10.3389/fbioe.2021.778496

**Published:** 2021-10-27

**Authors:** Yu-Jin Park, Dong-Myung Kim

**Affiliations:** Department of Chemical Engineering and Applied Chemistry, Chungnam National University, Daejeon, South Korea

**Keywords:** cell-free synthesis, protein production, horseradish peroxidase, heme, folding, prosthetic group, synthetic module, cofactor generation

## Abstract

One of the main advantages of a cell-free synthesis system is that the synthetic machinery of cells can be modularized and re-assembled for desired purposes. In this study, we attempted to combine the translational activity of *Escherichia coli* extract with a heme synthesis pathway for the functional production of horseradish peroxidase (HRP). We first optimized the reaction conditions and the sequence of template DNA to enhance protein expression and folding. The reaction mixture was then supplemented with 5-aminolevulinic acid synthase to facilitate co-synthesis of the heme prosthetic group from glucose. Combining the different synthetic modules required for protein synthesis and cofactor generation led to successful production of functional HRP in a cell-free synthesis system.

## Introduction

Although recombinant DNA technology has enabled heterologous production of recombinant proteins, many proteins require more than ordered polymerization of amino acids to achieve a functional state. For example, when expressing a eukaryotic gene in a microbial host cell, nascent polypeptides often fail to fold into functional structures unless assisted by appropriate foldases and other molecular chaperones (Baneyx and Mujacic, 2004; Rosano and Ceccarelli, 2014; Han et al., 2021; Ghag et al., 2021). In addition, many protein molecules require auxiliary chemical compounds for biological activity; most enzymes belonging to the E.C. 1 class (oxidoreductases) require organic prosthetic groups (Fruk et al., 2009). While functional production of those recombinant enzymes requires co-production of prosthetic groups, related synthetic pathways may be nonnative in host cells.

Due to its open nature, cell-free protein synthesis (CFPS) provides greater flexibility than cell-based gene expression methods. In particular, the biochemical conditions of protein synthesis can be readily manipulated so that the translational machinery can operate under the conditions designed to provide favorable environment for the production of functional target proteins. For instance, customizing the chemical composition of the reaction mixture for CFPS has enabled the production of many proteins that are difficult to be expressed in the cytoplasm of cells (Kim and Swartz, 2004; Carlson et al., 2012; Jin and Hong, 2018). In addition, CFPS can bypass the time-consuming and low-throughput procedures for cloning genes of interest into plasmid vectors (Ahn et al., 2005; Schinn et al., 2016). Although the early versions of CFPS systems suffered from low protein productivity and high reagent costs, recent advances in improving the energetics of ATP regeneration and methodologies of extract preparation have enabled the development of highly productive and economic CFPS systems (Kim et al., 2007; Jewett et al., 2008; Anderson et al., 2015; Lee and Kim, 2018). The CFPS systems employing crude cell extracts as the source of the translational machinery contain most of the soluble cytoplasmic enzymes, which can derive metabolic pathways that are activated by selective additions of substrates. For example, ATP regenerating metabolism can be activated when glucose or glycolytic intermediates are added to the reaction mixture for CFPS based on *Escherichia coli* (*E. coli*) extract (Kim and Swartz, 2001; Kim et al., 2007; Jewett et al., 2008). Thus far, however, studies on cell-free systems have mostly focused on the translation of genetic information into target proteins. Only a limited number of studies have been made on the use of selectively activated cell-free metabolic pathways for the generation of non-proteinous materials (Meyer et al., 2007; Schumperli et al., 2007; Bujara et al., 2011; Kwon et al., 2013).

In the present study, we used an *E. coli*-based CFPS system to produce functional horseradish peroxidase (HRP). Although HRP is a highly challenging target for recombinant production because functional HRP contains four intramolecular disulfide bonds and a properly assembled hemin prosthetic group, optimization of reaction components and parameters resulted in conditions that supported the synthesis of functional enzyme. The template DNA was also engineered to enhance the translation rate of HRP by adding a ubiquitin sequence in front of the HRP sequence. Finally, an enzymatic pathway for heme synthesis was incorporated into the reaction mixture optimized for the synthesis of apoHRP. The resulting enhanced synthesis of apoenzyme and the heme prosthetic group led to the successful production of functional HRP. Our results demonstrate the potential for integrating ribosomal protein synthesis with different biochemical modules to widen the application of CFPS.

## Materials and Methods

### Materials

ATP, GTP, UTP, CTP, CP, creatine kinase, *E. coli* total tRNA mixture, and restriction enzymes were purchased from Roche Applied Science (Indianapolis, IN, United States). _L_-[U-^14^C] leucine (11.9 GBq/mmol) was obtained from Amersham Biosciences (Uppsala, Sweden). 1-Step Ultra TMB-ELISA solution and HisProbe-HRP Conjugate were purchased from Thermo Fisher Scientific (Waltham, MA, United States). High-fidelity VELOCITY DNA polymerase was obtained from Bioline (London, United Kingdom). The *hemA* gene of *Rhodobacter capsulatus* (ATCC 11166) encoding 5-aminolevulinic acid synthase (ALAS) was synthesized by Twist Bioscience (San Francisco, CA, United States) after optimizing the codons for expression in *E. coli*. PCR primers listed in Supplementary Table S1 were synthesized by Macrogen (Seoul, Korea). *E. coli* S12 extracts were prepared from the BL21 Star (DE3) strain as described previously (Park et al., 2017). All other reagents were purchased from Sigma-Aldrich (St. Louis, MO, United States) and used without further purification.

### Preparation of Template DNA for Cell-Free Protein Synthesis

The *hrp* gene was cloned into the pET21a vector using *Nde*I/*Sal*I restriction sites to generate the pET21a-HRP construct. The template DNA for CFPS was prepared by PCR using a forward primer for the 15 base pairs upstream of the T7 promoter (T7Pro-15UP), and a reverse primer for the T7 terminator (T7Ter). The PCR mixture consisted of 1 ng/ µl template, 200 nM of each (forward and reverse) primer, 0.02 U/µl DNA polymerase, 1 mM each of dNTP, and 1 × Hi-Fi buffer (Bioline). Thermal cycling involved heating at 95°C for 2 min, followed by 28 cycles at 95°C for 30 s, 55°C for 1 min, 72°C for 2 min, and a final extension at 72°C for 2 min. PCR products were purified using a PCR Clean-up kit (Promega, Madison, WI, United States) prior to use for CFPS. For randomization of the +2 and +3 codons, the *hrp* gene was amplified using a forward primer containing degenerated +2 and +3 codons (+2/+3MutHRP, Supplementary Table S1), re-cloned into the pET21a plasmid, and transformed into competent *E. coli* DH5α cells. After overnight culturing of the transformed *E. coli* DH5α cells on Luria-Bertani (LB) agar plates, individual colonies were picked for colony PCR to prepare template DNA for CFPS.

### Cell-free Synthesis of Horseradish Peroxidase

The standard reaction mixture for cell-free synthesis of HRP and ubiquitin-fused HRP (ubi-HRP) consisted of 57 mM HEPES-KOH (pH 8.2); 1.2 mM ATP; 0.85 mM each of CTP, GTP, and UTP; 0.17 mg/ml *E. coli* total tRNA mixture (from strain MRE600); 0.64 mM cAMP; 90 mM potassium glutamate; 80 mM ammonium acetate; 12 mM magnesium acetate; 34 μg/ml _L_-5-formyl-5,6,7,8-tetrahydrofolic acid; 2 mM each of the 20 essential amino acids; 10 μM _L_-[U-^14^C]leucine (11.9 GBq/mmol); 2% (w/v) polyethylene glycol-8000; 67 mM creatine phosphate; 3.2 µg/ml creatine kinase; 2 mM calcium acetate; 67.7 µM hemin; 5 mM reduced glutathione (GSH); 5 mM oxidized glutathione (GSSG); 13.3 ng/µl template DNA; and 26% (v/v) S12 extract. A mixture of S12 extract and ubiquitin hydrolase (UBP1)-enriched S12 extract (3:1 v/v) was used in experiments involving *in situ* cleavage of ubiquitin during the synthesis of ubi-HRP. Cell-free synthesis reactions were performed for 3 h at different temperatures.

### Analysis of Cell-free Synthesized Horseradish Peroxidase

The amount of cell-free synthesized protein was determined by measuring trichloroacetic acid (TCA)-precipitated radioactivity of the reaction mixture as described previously (Byun et al., 2019). The amount of soluble protein was determined by measuring the TCA-precipitated radioactivity of the supernatant after centrifugation (10,000 × *g*, 10 min) of the reaction mixture. For western blot analysis to determine the size of synthesized protein, poly-histidine tag was added to the C-terminus of HRP. HisProbe-HRP was used for direct detection of poly-histidine tagged proteins following the manufacturer’s instruction. Enzymatic activity of HRP was determined using 1-Step Ultra TMB-ELISA solution, which contains 3,3’,5,5’-tetramethylbenzidine (TMB) and hydrogen peroxide. After diluting the completed reaction mixture with 10 volumes of phosphate-buffered saline (PBS), 2 µl of the diluted solution was mixed with 50 µl TMB substrate solution. After a 3 min incubation at room temperature, 50 µl of 1 M sulfuric acid was added to quench the chromogenic reaction, and the absorbance was measured at 450 nm using a Clariostar microplate reader (BMG Labtech, Ortenberg, Germany).

### Preparation of Purified Aminolevulinic Acid Synthase

The *hemA* gene was cloned into the pET28a plasmid using the *Nco*I/*Xho*I restriction sites to generate the pET28a-ALAS construct. For preparation of ALAS, *E. coli* strain BL21 Star (DE3) cells transformed with the pET28a-ALAS plasmid were cultured in 200 ml LB medium supplemented with kanamycin (25 mg/L). Isopropyl β-D-1-thiogalactopyranoside (1 mM) was added to induce the expression of ALAS after cells had grown to an absorbance at 600 nm (OD_600_) of 0.6. Cells were harvested at OD_600_ = 4, and disrupted with a French Press cell at 12,000 psi after being resuspended in lysis buffer (50 mM NaH_2_PO_4_, 300 mM NaCl, 10 mM imidazole, pH 8.0). The supernatant of the lysate resulting from centrifugation (15,000 × *g* for 30 min at 4°C) was loaded onto a 5 ml Ni-NTA agarose bead column (Qiagen, Hilden, Germany). After washing four times with 10 ml washing buffer (50 mM NaH_2_PO_4_, 300 mM NaCl, 30 mM imidazole, pH 8.0), ALAS was eluted with 2 ml elution buffer (50 mM NaH_2_PO_4_, 300 mM NaCl, 250 mM imidazole, pH 8.0). The eluate was dialyzed against 2 L PBS using Spectra/Por 2 dialysis tubing (molecular weight cut-off: 12–14 kDa) according to the manufacturer’s instructions (Repligen, Waltham, MA, United States). The purity of ALAS was determined by 16% tricine-sodium dodecyl sulfate-polyacrylamide gel electrophoresis followed by Coomassie Blue staining (Supplementary Figure S3). The concentration of purified ALAS was measured by Bradford assay (Bradford, 1976).

## Results and Discussion

### Optimization of Reaction Conditions for Cell-free Synthesis of Horseradish Peroxidase

**FIGURE 1:**
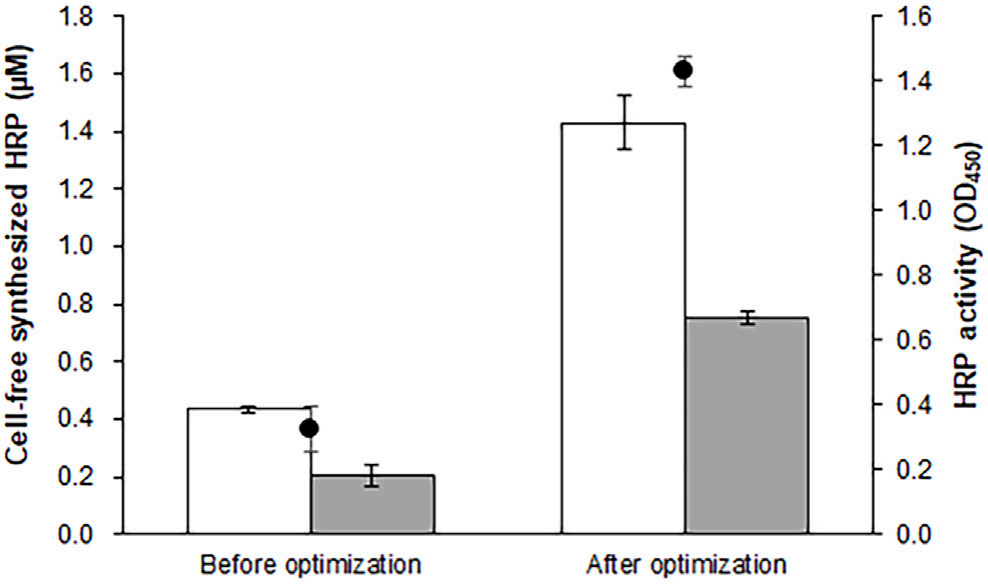
Optimization of reaction conditions for cell-free synthesis of HRP. The reaction mixture was optimized for the synthesis of functional HRP. The graph compares the total and soluble yields (blank and filed bars, respectively) and enzyme activity (circles) of HRP produced under the reaction conditions before and after the optimization. Measurements were performed in triplicate, and the error bars represent the standard deviations of three independent experiments.

Peroxidases catalyze redox reactions on a wide range of substrates, and have potential industrial and biotechnological applications (Regalado et al., 2004; Lopes et al., 2014; de Oliveira et al., 2021). HRP C1A (hereafter referred to as HRP), the major enzyme among the 19 isoenzymes produced in horseradish root (Krainer and Glieder, 2015), is one of the most extensively studied peroxidases. Comprising 308 amino acids, this enzyme contains a hemin prosthetic group and two Ca^2+^ ions. It also requires four sets of properly formed disulfide bridges for full biological activity (Veitch, 2004). When expressed in *E. coli* cells, recombinant HRP forms inclusion bodies, and requires time- and labor-intensive refolding steps to recover the functional enzyme (Chaunhan and Kang, 2018). Similarly, incubation of template DNA in the standard reaction mixture produced ∼0.4 μM (16 μg/ml) of inactive protein (Figure 1). Taking advantage of the open nature of CFPS, we attempted to adjust the molecular environment of the translation reaction to achieve expression of functional HRP. Assuming that inefficient formation of disulfide bonds was one of the factors preventing the synthesis of functional HRP, we replaced the S12 extract with one enriched with DsbA and DsbC (Schlapschy et al., 2006). As expected, this led to the generation of significantly increased peroxidase activity in the reaction mixture (Supplementary Figure S1A). It was also found that expression of functional HRP was heavily dependent on the reaction temperature of cell-free synthesis: optimizing the reaction temperature markedly enhanced HRP activity (Supplementary Figure S1B). Although a lower temperature generally reduces the overall yield of protein synthesis, HRP activity in the reaction mixture increased 3-fold when the reaction temperature was shifted from 30°C to 20 °C. The concentrations of calcium, hemin, and glutathione were subsequently optimized to maximize the yield of functional HRP (Supplementary Figures S1C−E). The optimized reaction conditions (S12 extract enriched with DsbA/C, 20 °C reaction temperature, 0.5 mM GSH, 0.5 mM GSSG, 16.7 mM hemin, 1.0 mM calcium acetate) were similar to those reported previously by Zhu et al. (2015). Under these conditions, ∼57% synthesized HRP was partitioned in the soluble fraction and exhibited 4-fold higher peroxidase activity than enzyme produced under standard reaction conditions (Figure 1).

### Engineering the Template DNA Enhances Expression of Horseradish Peroxidase

**FIGURE 2:**
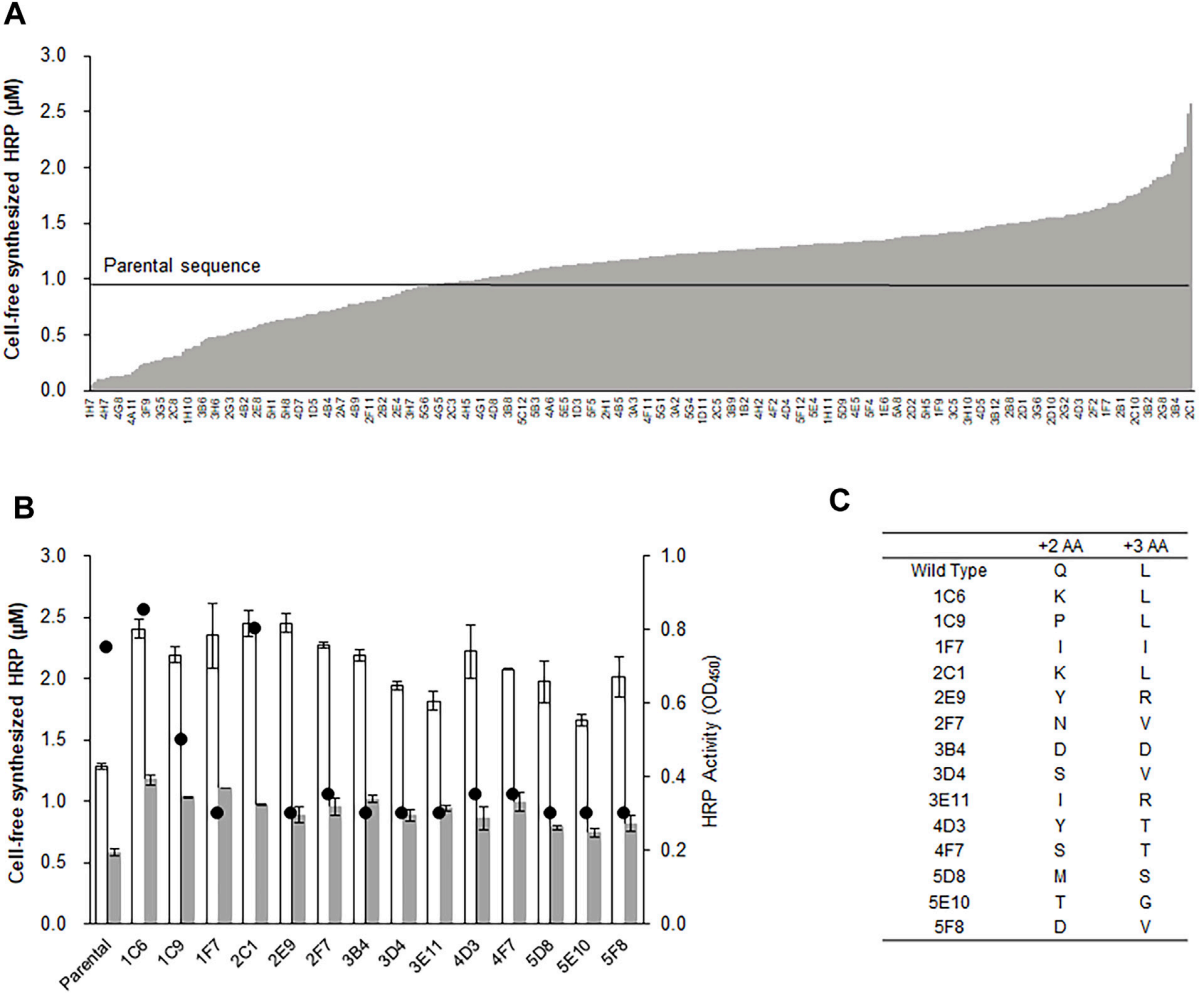
Randomization and selection of initial codons for enhanced production of HRP. The +2 and +3 codons of the hrp gene was randomized by PCR using degenerate primers. After transforming *E. coli* cells with the library of the plasmids containing randomized genes, each of the variant genes were amplified by colony-PCR, and expressed in the optimized reaction mixture for cell-free protein synthesis. **(A)** Resulting yields of HRP synthesis from the individual variant genes. The labels of X-axis indicate the locations of variant clones in 96 well plates. **(B)** Total yields (blank bars), soluble yields (filled bars), and enzyme activity (circles) of selected clones. Measurements were performed in triplicate, and the error bars represent the standard deviations of three independent experiments. **(C)** Amino acid residues at the +2 and +3 codons of the variant clones.

After optimizing the physicochemical reaction parameters, we attempted to enhance the production of HRP by engineering the nucleotide sequence of the template DNA. It has been reported that the efficiency of protein translational is strongly influenced by the nucleotide sequences of the initial codons downstream of the start codon. For example, when nucleotides were randomly changed at the +2 and +3 codons of human erythropoietin (hEPO), several variant genes exhibited expression yield increases by as much as 7-fold (Ahn et al., 2008). Based on this, we randomized the +2 and +3 codons of the HRP gene and measured the relative expression levels of 500 variant clones. As expected, the variant HRP genes exhibited a wide distribution of expression levels, some of which were substantially higher than the parental sequence (Figure 2A). Interestingly, subsequent enzyme activity assays revealed that expression levels of some variants did not always correlate with enzymatic activity. For example, while the 1F7 variant exhibited more than a 2-fold increase in HRP production, the peroxidase activity of this variant was less than 40% that of the parental gene. In total, 15 out of 500 variant clones showed enhanced expression levels, but only two exhibited enzyme activity similar to that of the parental gene (Figure 2B). Sequencing of the variant genes revealed that it is critical for variant enzymes to encode leucine in the +3 codon for HRP activity. The three clones exhibiting significant activity were found to have synonymous +3 leucine codons, and all other clones had substituted codons at the same position (Figure 2C). Moreover, in a separate experiment in which the +3 position was replaced with the other 19 essential amino acids, even conservative replacements (glycine, alanine, and valine) failed to support HRP activity (Supplementary Figure S2A). In addition to the +3 position, the identity of the codon at the +2 position also appeared to affect HRP activity because the three clones in which the +2 codon was changed to lysine or proline from glutamine exhibited comparable or lower activity, although the expression levels were enhanced by almost 2-fold. Because these N-terminal residues are not in close proximity to the cofactor pockets or active site (Supplementary Figures S2B,C), it seems reasonable to infer that the leucine residue at this position plays an important role in forming the active structure of HRP (Yates et al., 2014; Kelly et al., 2015).

### Expression and Co-translational Cleavage of a Translation-Enhancing Fusion Partner

**FIGURE 3:**
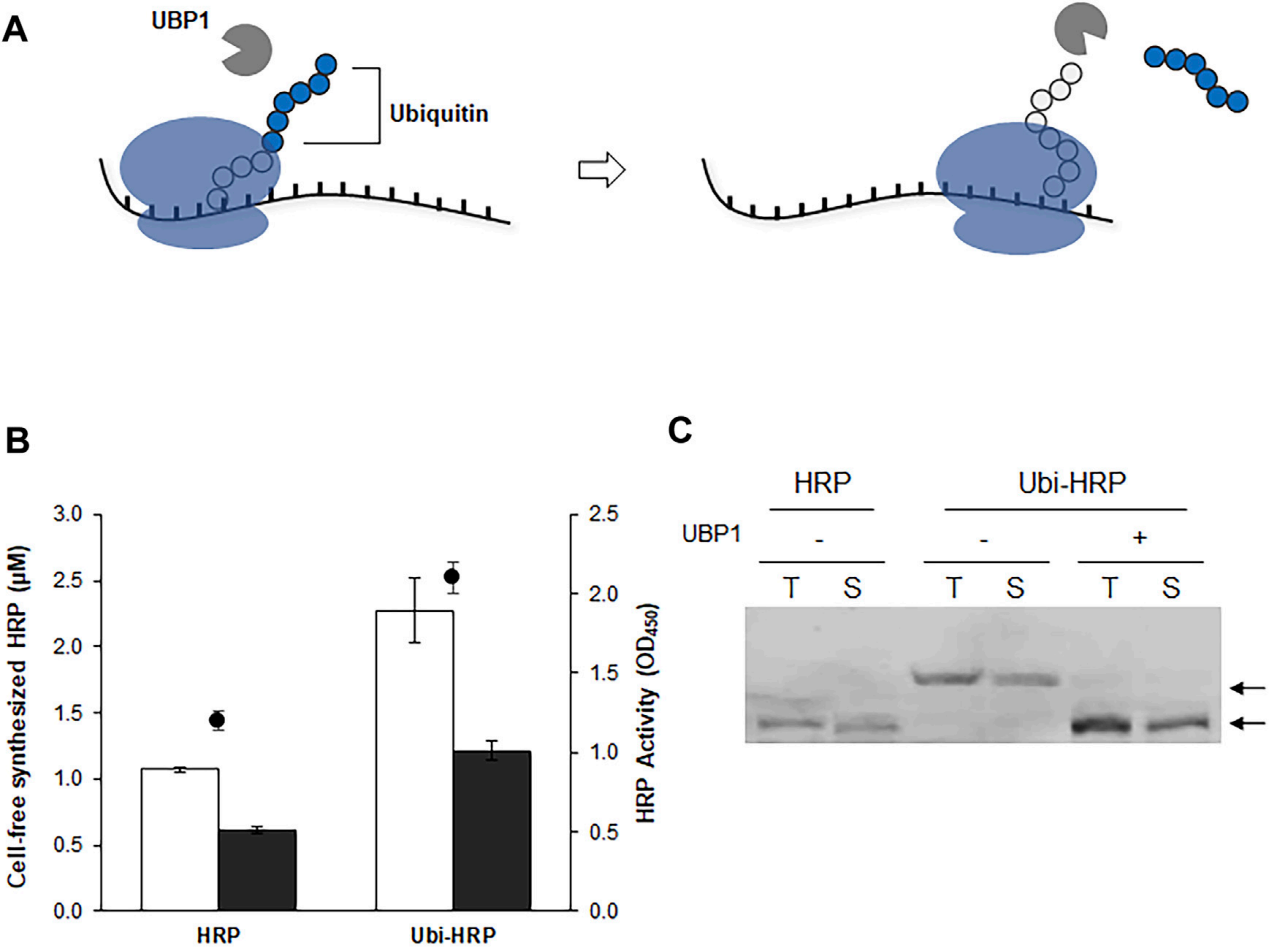
Enhanced translation and *in situ* cleavage of ubiquitin-tagged HRP. **(A)** The template DNA is fused with a ubiquitin sequence, which enhances the cell-free production of HRP. Due to the presence of UBP1 in the cell extract, the ubiquitin sequence is cleaved off the translation product, enabling the synthesis of HRP in its native amino acid sequence. **(B)** Total yields (blank bars), soluble yields (filled bars), and enzyme activity (circles) of the cell-free synthesized HRP. **(C)** Western blot analysis of cell-free synthesized HRP. Arrows indicate the molecular weights of HRP in the presence (Ubi-HRP) or absence (HRP) of the N-terminal ubiquitin tag.

The results described above indicate that changing the initial codons is not a valid option for enhancing the expression of functional HRP. We therefore took an alternative approach by introducing an N-terminal fusion partner. N-terminal fusion partners are often used to boost the translation efficiency and increase the solubility of recombinant proteins (Ahn et al., 2011). However, we were concerned that the presence of a fusion partner might affect the enzyme activity of HRP, which is sensitive to the amino acid residue at the N-terminus. Previously, we demonstrated that a ubiquitin sequence can be used as an effective N-terminal fusion partner to enhance the translational efficiency and solubility of recombinant proteins that are otherwise difficult to produce in significant yields (Kasi et al., 2017). One of the advantages of using ubiquitin is that it has a cognate protease, ubiquitin hydrolase (UBP1), which removes ubiquitin without leaving additional amino acid residues on the cargo protein (Figure 3A). Therefore, by using a UBP1-enriched extract, a CFPS reaction can produce a target protein with its native N-terminal amino acid sequence from a template DNA fused to the ubiquitin gene. This approach was successfully employed to enhance the cell-free synthesis of HRP. In the presence of the N-terminal ubiquitin sequence, the expression level of HRP was increased more than 2-fold (Figure 3B). Western blotting analysis revealed that most of the synthesized protein was the correct size for HRP, indicating successful co-translational cleavage of ubiquitin by the UBP1 enzyme (Figure 3C). Therefore, in subsequent experiments, the ubiquitin-fused HRP (ubi-HRP) construct was used in combination with UBP1-enriched S12 extract to achieve enhanced production of HRP with its native N-terminal amino acid sequence.

### Co-Synthesis of Apo-Enzyme and the Heme Prosthetic Group for One-Pot Generation of Functional Horseradish Peroxidase

**FIGURE 4:**
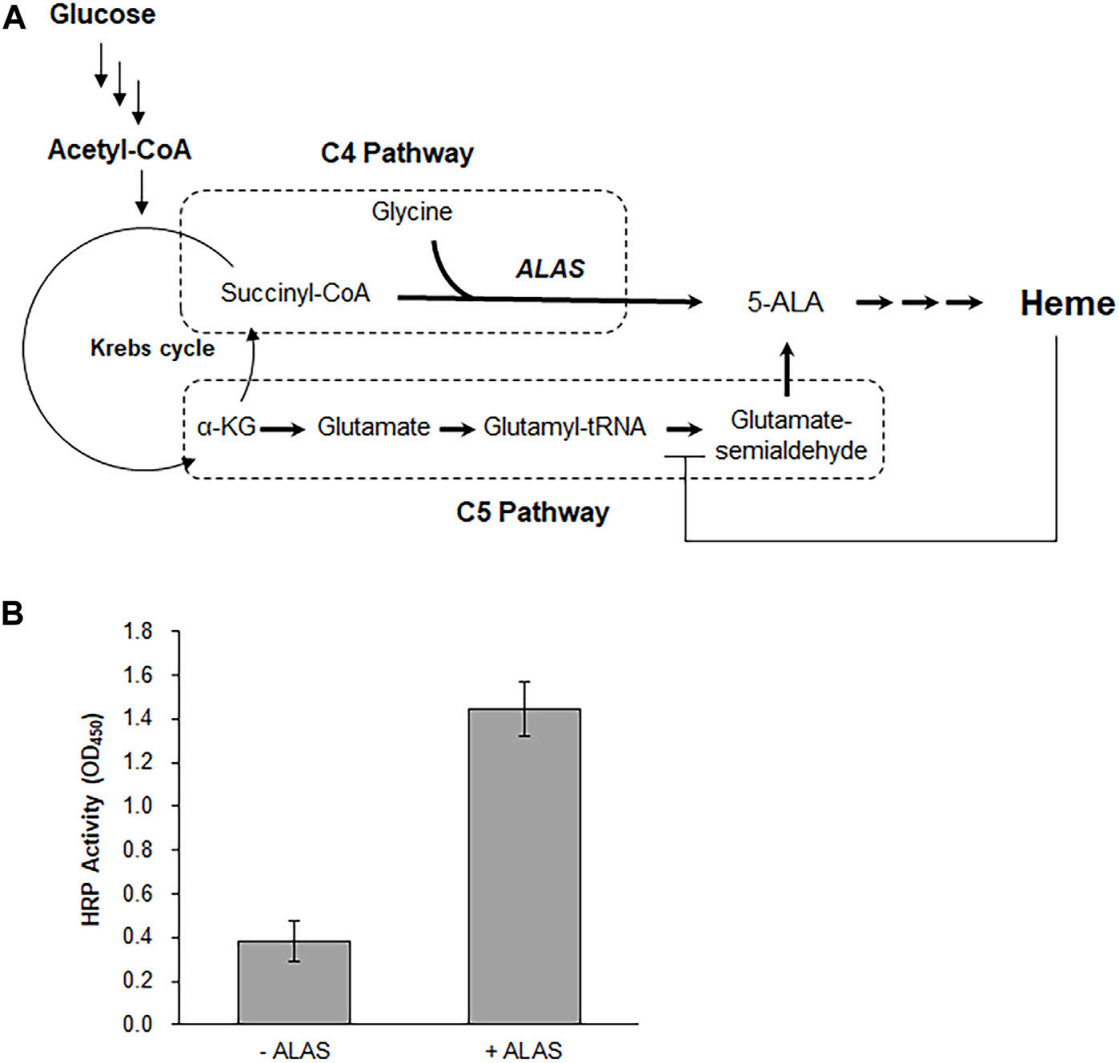
Co-synthesis of heme and apo-HRP. **(A)** To activate the C4 pathway for the synthesis of heme from glucose, the reaction mixture was supplemented with purified ALAS from *R. capsulatus*. **(B)** Effect of ALAS addition on the enzyme activity of cell-free synthesized HRP.

Because the cell-free synthesis reaction applied in this study employed a crude cell extract, it is reasonable to assume that most of the metabolic enzymes of *E. coli* are retained in the reaction mixture. Taking advantage of this, we attempted to provide the CFPS system with an *in situ* supply of the heme prosthetic group. In *E. coli*, cells use the C5 pathway for heme synthesis, and the generation of 5-aminolevulinic acid (5-ALA) is tightly regulated via inhibition of glutamyl-tRNA reductase by heme (Woodard and Dailey, 1995) (Figure 4A). Therefore, to create a sufficient pool of 5-ALA from glucose, we activated the alternative C4 pathway by introducing the ALAS from *Rhodobacter capsulatus* (*R. capsulatus*). In the C4 pathway, 5-ALA is generated from succinyl-CoA and glycine. Since the Krebs cycle is operational in *E. coli* extracts, we predicted that feeding glucose to the cell-free synthesis system would generate succinyl-CoA, which can be condensed with glycine in the reaction mixture to supply 5-ALA for subsequent heme synthesis (Kwon et al., 2013). As expected, cell-free expression of the ubi-HRP gene in the reaction mixture supplemented with 250 µg/ml ALAS, 180 mM glucose, and 2.5 mM glycine resulted in the production of functional HRP (Figure 4B and Supplementary Figure S4).

## Conclusion

The complex structure of HRP makes it a difficult target for bacterial recombinant protein production. When expressed in the cytoplasm of *E. coli*, HRP forms inactive inclusion bodies containing trace amounts of the heme prosthetic group. In the present study, we demonstrated that the open nature and modularity of the cell-free synthesis system can be harnessed for the production of functional HRP by the bacterial translational machinery. In addition to modifying the reaction conditions to facilitate proper folding of HRP, the template DNA was engineered to enhance the translational efficiency of the HRP gene. The improved conditions for the synthesis of HRP apoenzyme were then combined with the C4 pathway for *in situ* generation of the heme prosthetic group. Combining these different synthetic modules achieved efficient expression of functional HRP in a cell-free synthesis system derived from *E. coli* extract.

## Data Availability

The original contributions presented in the study are included in the article/Supplementary Material, further inquiries can be directed to the corresponding author.
