# Supplementary material for: Production of Recombinant Horseradish Peroxidase in an Engineered Cell-free Protein Synthesis System
Source: Front Bioeng Biotechnol. 2021 Oct 27;9:778496. doi: 10.3389/fbioe.2021.778496 (PMC8579056; doi:10.3389/fbioe.2021.778496)
Supplement: Supplementary file 1 [file DataSheet1.docx]

**Supplementary Information**

Production of recombinant horseradish peroxidase in an engineered cell-free protein synthesis system

**Running title: Cell-free production of HRP**

Yu-Jin Park and Dong-Myung Kim^*^

Department of Chemical Engineering and Applied Chemistry, Chungnam National University, Yuseong-gu, Daejeon 34134, Korea

**^*^Correspondence:**

Dong-Myung Kim

[dmkim@cnu.ac.kr](mailto:dmkim@cnu.ac.kr)


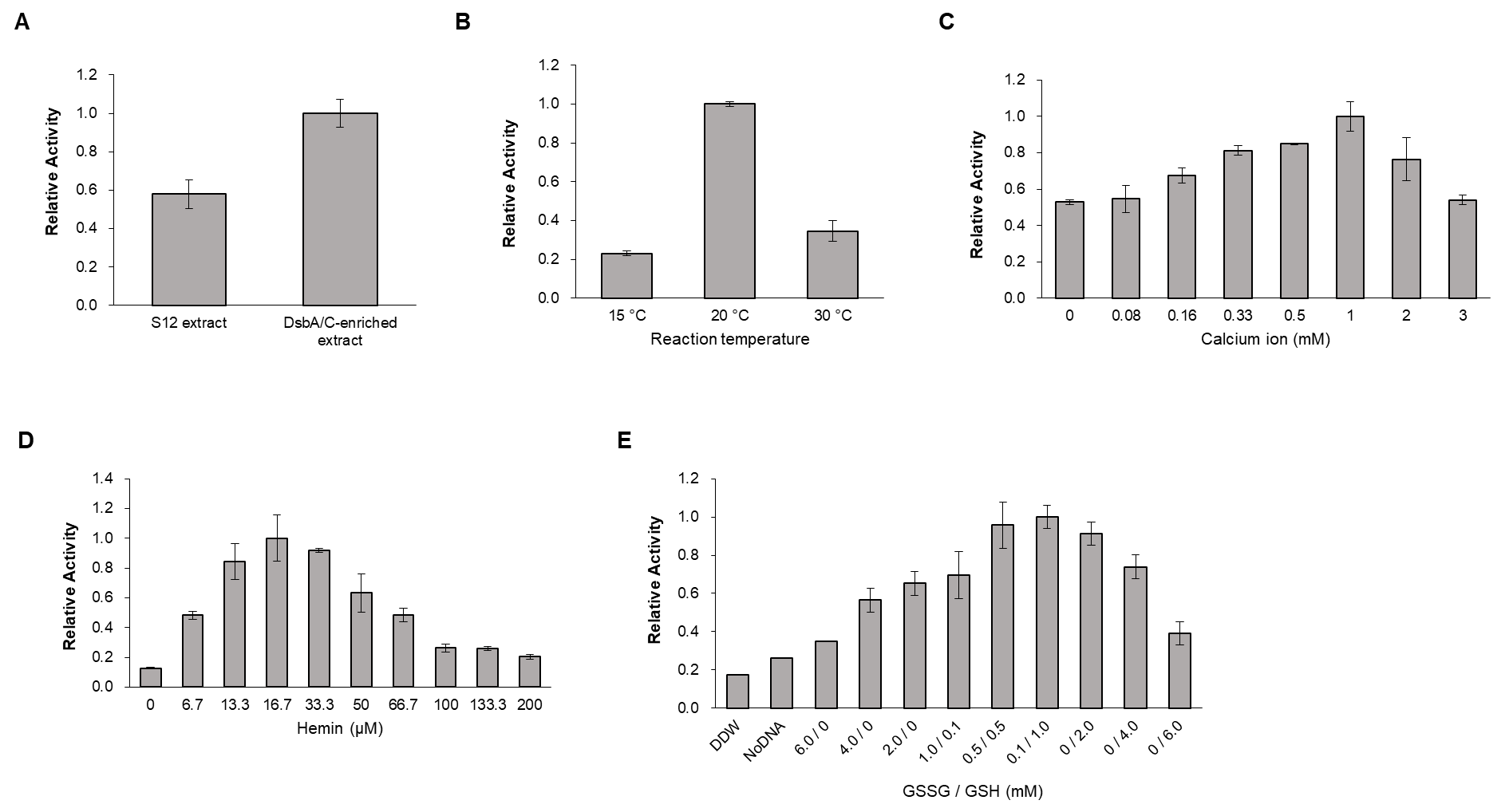


**Figure S1. Optimization of reaction conditions for cell-free synthesis of HRP.**

**
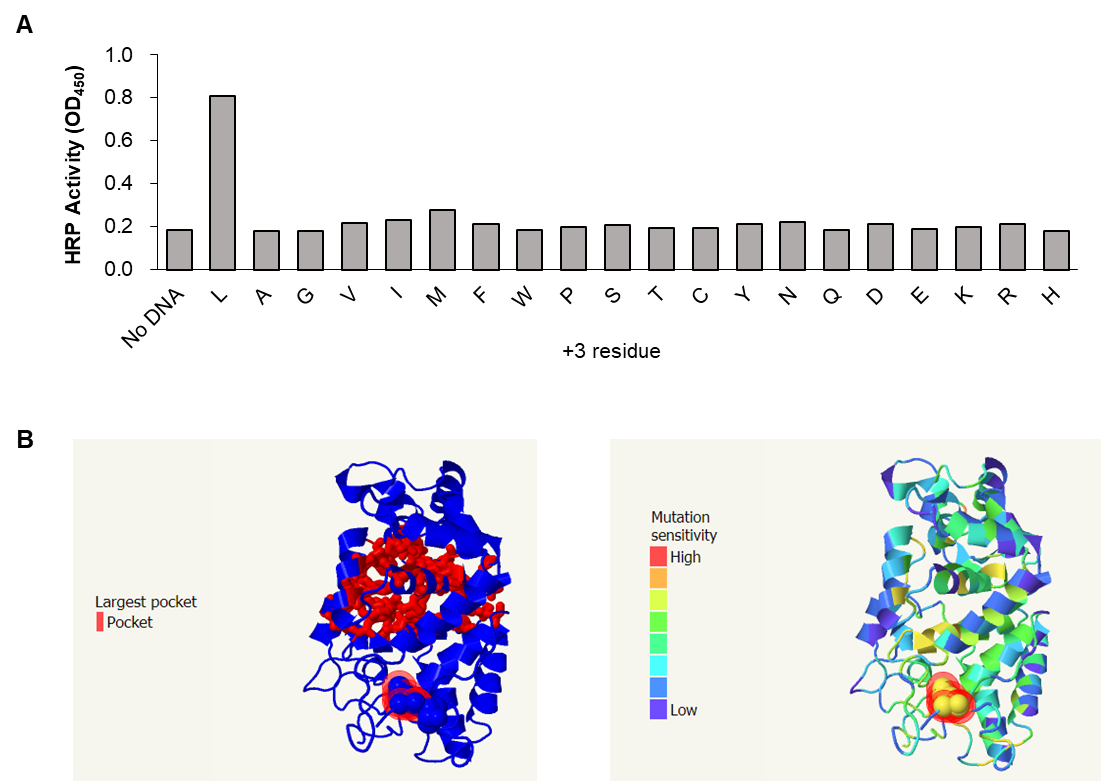
**

**Figure S2. Effect of the third codon on the enzyme activity of recombinant HRP.** (A) Dependence of HRP activity on the identity of the +3 residue. (B) Structural analysis of HRP using Phyre2 (<http://www.sbg.bio.ic.ac.uk/phyre2/html/page.cgi?id=index>). Active pocket of HRP is shown in the left image. Predicted mutation sensitivity of a particular position is shown in the right image. Red circles in the structure indicate leucine at the third residue position of HRP.

**
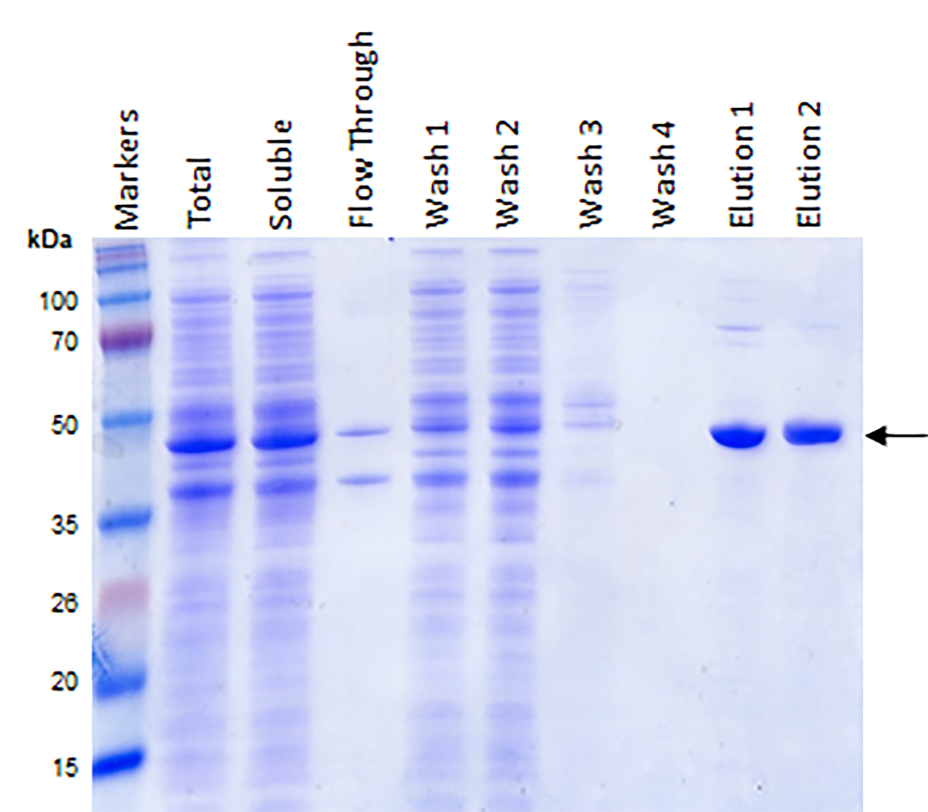
**

**Figure S3. SDS-PAGE analysis of purified ALAS.** ALAS was expressed in *E. coli* cells, and purified using a Ni-NTA column. 5 µL of each fraction was mixed with 2 × SDS sample buffer, and heated for 5min at 95°C. 5 µL of the heat-treated sample was loaded onto 16% SDS-PAGE gel. Arrow indicates the recombinant ALAS (~45kDa).


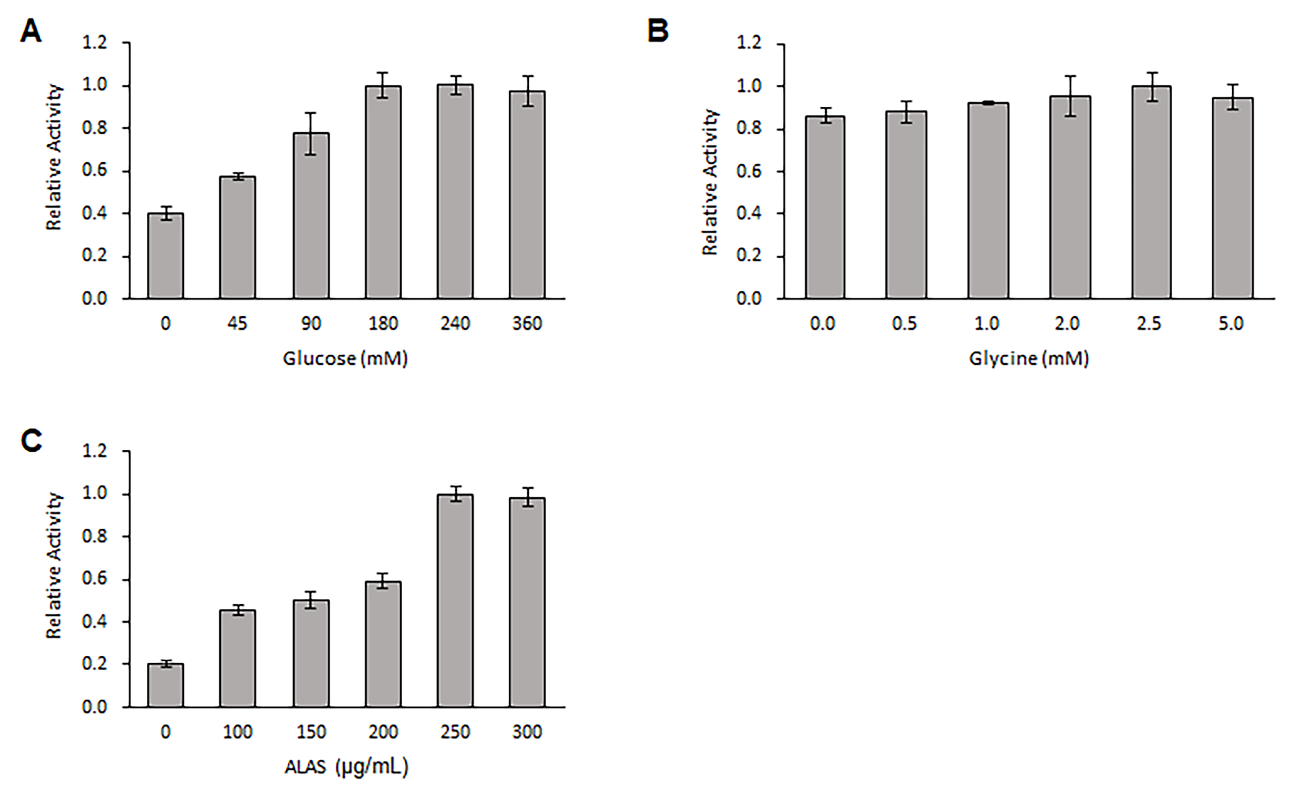


**Figure S4. Optimization of the concentrations of glucose, glycine, and ALAS.**

**Table S1. Primer sequences used for PCR**

| **Primer name** | **Sequence (5’ to 3’)** |
| --- | --- |
| T7Pro-15UP  T7Pro-200UP  T7Pro-Rev  T7Ter  +2/+3MutHRP^*^ | TCGATCCCGCGAAATTAATACGACTCACTATAGG  TGGCGCCCAACAGTC  CATATGTATATCTCCTTCTTAAAGTTAAACAAAATTATTTCTAGAG  CAAAAAACCCCTCAAGACCCGTTTA  GTTTAACTTTAAGAAGGAGATATACATATGNNNNNNACCCCGACGTTTTATGATAACAGCTGTCCC |

**^*^**Degenerate primer used for randomizing the +2/+3 codons of HRP
